# Improve Intermetal Dielectric Process for HTRB Stability in Power GaN High Electron Mobility Transistor (HEMT) by unbiased-Highly Accelerated Stress Testing (uHAST)

**DOI:** 10.3390/mi16111233

**Published:** 2025-10-30

**Authors:** Yu-Ting Chuang, Niall Tumilty, Tian-Li Wu

**Affiliations:** 1 MossFloat Ltd., Hsinchu City 30010, Taiwan; 2 International College of Semiconductor Technology, National Yang Ming Chiao Tung University, Hsinchu City 30010, Taiwan; ntumilty@nycu.edu.tw; 3 Institute of Electronics, National Yang Ming Chiao Tung University, Hsinchu City 30010, Taiwan

**Keywords:** p-GaN HEMTs, dielectric delamination, HTRB, uHAST

## Abstract

This study investigates a severe high-temperature reverse bias (HTRB) failure observed in GaN HEMTs, with devices failing in under 24 h. We conducted an in-depth analysis of the electrical and physical failure mechanisms, revealing that unbiased-highly accelerated stress testing (uHAST) can effectively induce dielectric delamination. The electrical and physical characteristics of devices post-delamination demonstrated a strong correlation between delamination at the nitride–polyimide interface and an increase in off-state drain leakage current (I_DSS_). Our findings led to the removal of a suspected process step involving the use of the reactive chemical, N-methyl-2-pyrrolidone (NMP), before and after polyimide deposition. This critical process change yielded a significant improvement in reliability; while the initial failure rate was 25% at 24 h, three lots of 260 parts subsequently survived 1000 h of HTRB stress with no failure. In conclusion, uHAST is a valuable reliability testing tool for assessing package and film adhesion, leveraging high pressure and moisture to quickly identify and troubleshoot delamination-related reliability issues.

## 1. Introduction

Gallium nitride, commonly referred to as GaN, is a type of wide-bandgap semiconductor material. This material has several advantages over traditional silicon-based semiconductor materials, particularly when it comes to power density and efficiency [1]. Compared to traditional silicon metal–oxide semiconductor field-effect transistors (MOSFETs) and insulated gate bipolar transistors (IGBTs), GaN enables the creation of more efficient and compact power systems [2].

One of the key benefits of GaN is its ability to process power more efficiently than silicon-only solutions. This results in a significant reduction in power loss. Additionally, the increased efficiency of GaN reduces the need for added cooling components, which can further simplify system design [3,4].

The increased power density of GaN also enables the creation of smaller, lighter systems. By packing more power into smaller spaces, designers can create more compact and efficient systems that are better suited to a wide range of applications [5,6]. Overall, the advantages of GaN make it an attractive material for a variety of power electronics applications.

Building upon the advantages of GaN devices, our goal is to develop reliable GaN devices that meet the stringent standards set by the Joint Electron Devices Engineering Council (JEDEC). Under high-temperature reverse bias (HTRB) stress, a positive shift in ON resistance (R_DSON_) and threshold voltage (V_TH_) is commonly reported, although these changes do not typically lead to a failed state. In more accelerated HTRB experiments, where the drain voltage exceeds the rated value, some early GaN HEMTs have shown a V_TH_ shift of up to 50%, which exceeds the notional 20% failure boundary but has been found to be recoverable. These observed shifts in electrical parameters across both device types are all attributed to underlying electron trapping and detrapping mechanisms [7,8].

While numerous research papers have explored the characteristics of GaN devices prior to metal routing, we would like to shift the focus to a critical aspect that has received less attention: the impact of process excursions during metal routing on the reliability of GaN devices, particularly in relation to high-temperature reverse bias (HTRB) failures.

In other words, we aim to investigate how variations in the metal routing process can affect the reliability of GaN devices, leading to HTRB failures. By examining this critical stage of the manufacturing process, we hope to gain a deeper understanding of the factors that influence the reliability of GaN devices and identify opportunities for improvement.

## 2. Materials and Methods

**Figure 1:**
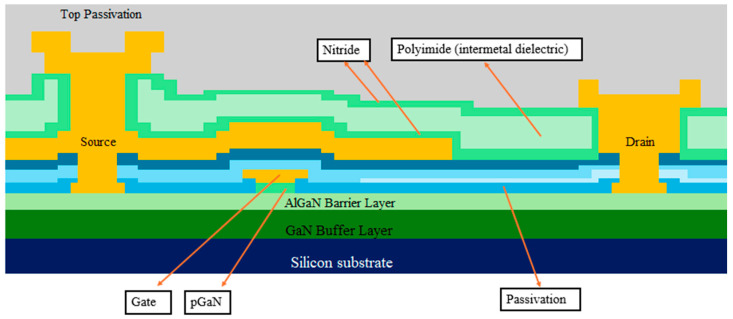
A cartoon structure of a typical power GaN HEMT.

**Figure 2:**
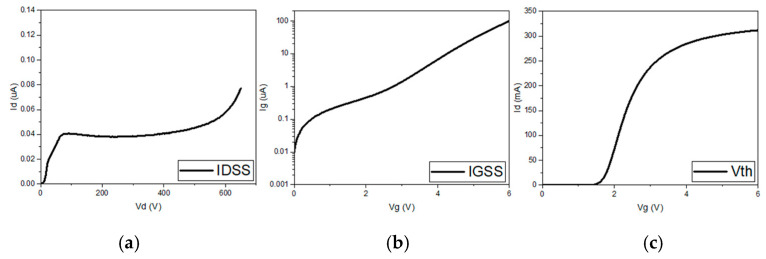
Typical (**a**) I_d_-V_d_ at V_d_ = 0 V to 650 V and V_g_ = 0 V, (**b**) I_g_-V_g_ at V_g_ = 0 V to 6 V and V_d_ = 0 V (**c**) I_d_-V_g_ at V_d_ = 0.1 V curves.

The cross-section of typical commercial p-GaN power HEMTs is shown in Figure 1. The epitaxial structure consists of a super-lattice GaN buffer layer, an AlGaN barrier layer and a pGaN layer. Figure 2 shows the typical I-V curve of the GaN HEMTs.

## 3. Results and Discussion

**Figure 3:**
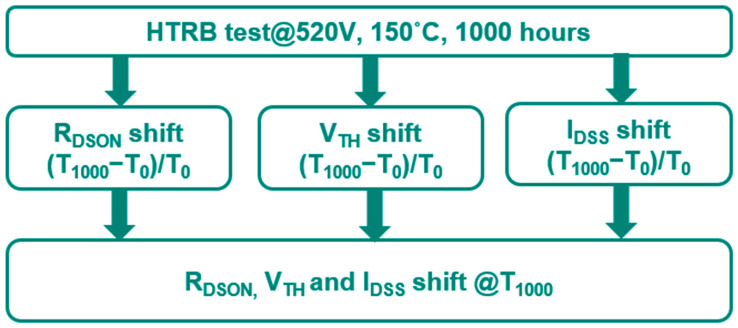
HTRB test comparison for typical power GaN HEMT.

To verify the device reliability, HTRB is performed under V_d_ = 520 V, 150 °C and 1000 h as shown in Figure 3. The R_DSON_, V_TH_ and I_DSS_ shifts are critical parameters for the HTRB test.

**Figure 4:**
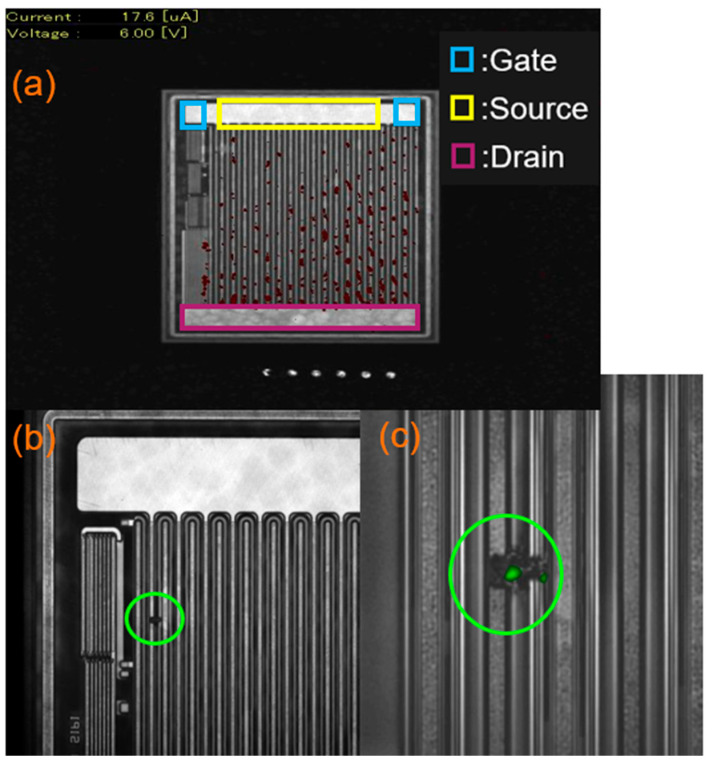
InGaAs hot spot failure analysis. (**a**) Top metal delayered with InGaAs hot spot overview. (**b**) A high-leakage hot spot with failure between the drain and field plate at I_d_ = 6 V and V_g_ = 0 V. (**c**) Zoomed-in image of (**b**) with catastrophic failure. Hot spots are small in red for (**b**) and green for (**c**). It is better to be identified with green circle.

One of the failed devices exhibited a catastrophic increase in drain leakage current, I_DSS_, after only 24 h of the HTRB stress test, indicating a short circuit failure mode. To comprehensively investigate this anomaly, decapsulation and hot spot analysis utilizing thermal techniques were performed, as summarized in Figure 4.

The underlying device utilizes indium gallium arsenide (InGaAs), a III-V semiconductor valued for its superior electron mobility and advantageous properties. However, these same characteristics render InGaAs components susceptible to “hot spots”—discrete regions of excessive thermal generation. These anomalies are fundamentally caused by localized current crowding or intense electric field peaks, which dramatically increase power dissipation within a constrained volume. Such hot spots are often invisible to the naked eye, necessitating high-resolution thermal imaging for precise identification.

To enhance visibility, the top metal layer was delayered. The analysis revealed that the majority of hot spots were localized within the active cell area, as shown in Figure 4a. While most thermal anomalies were observed between the drain and source terminals, one specific critical hot spot was detected between the drain and the field plate. This particular drain-to-field-plate failure site was selected for detailed examination, as shown in Figure 4b. The magnified top-view image in Figure 4c definitively confirms a severe burnout, leading to a subsequent focused ion beam (FIB) cross-section to elucidate the physical failure mechanism.

**Figure 5:**
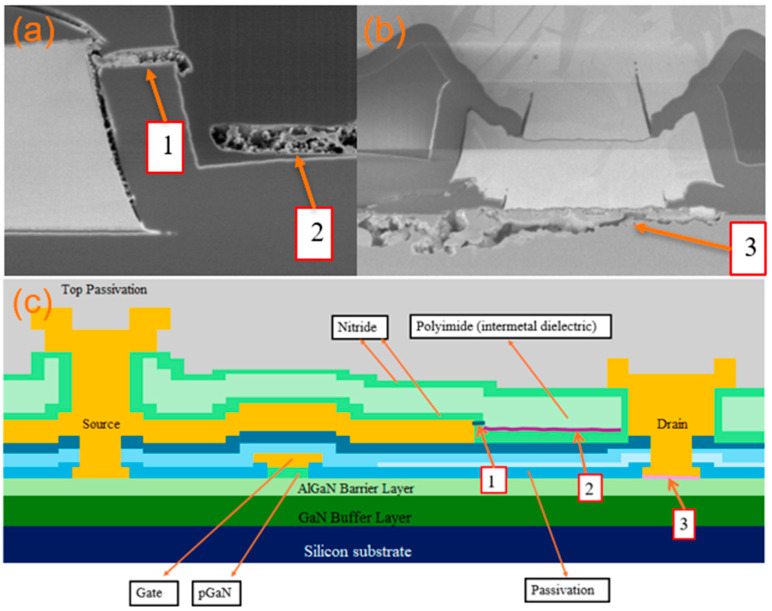
FIB of Figure 4c hot spot. (**a**) Cross-section of findings 1 and 2. (**b**) Cross-section of finding 3. (**c**) Cartoon illustration of corresponding films and functions.

The FIB cross-section in Figure 5 is from the hot spot that was identified in Figure 4c. There are many cracks and delamination as shown in Figure 5a and burnout as shown in Figure 5b. A cartoon illustration in Figure 5c helps to simplify and identify the findings. Finding 1 is a nitride crack, finding 2 is intermetal dielectric polyimide delamination and finding 3 is drain burnout in Figure 5c. Finding 1 might be due to thermal accumulation from HTRB overcoming the stress from the nitride film, causing a crack. Finding 2 might be due to chemical residue that reacts with the dielectric, causing an increase in the device leakage current with heat dissipation, leading to delamination. Finding 3 might be due to high leakage current from the drain causing local burnout. To further clarify the root cause, the uHAST is introduced to test the dielectric stability.

**Figure 6:**
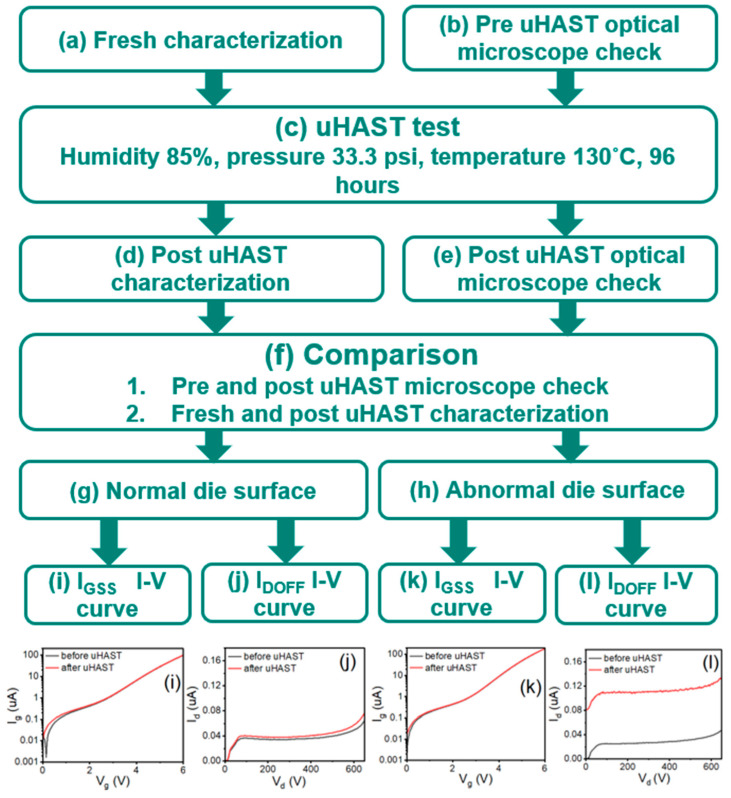
Unbiased highly accelerated stress test (uHAST) flow. (**a**) Characterize the standard I-V curve with fresh wafer. (**b**) Check with the optical microscope for die surface abnormity before uHAST. (**c**) Standard highly accelerated stress test (uHAST) from JEDEC. (**d**) Characterize the standard I-V curve after uHAST. (**e**) Check with the optical microscope for die surface abnormity after uHAST. (**f**) Make comparison of the optical microscope check before and after uHAST. Cross check the characterization of I_GSS_ and I_DSS_ between fresh and post-uHAST wafer. (**g**) Find the dies with normal surface for the follow-up characterization comparison. (**h**) Find the dies with abnormal surface for the follow-up characterization comparison. (**i**) Standard I_GSS_ I-V curve at V_g_ = 0 V to 6 V and V_d_ = 0 V before and after uHAST with normal die surface. (**j**) Standard I_DSS_ I-V curve at V_d_ = 0 V to 650 V and V_g_ = 0 V before and after uHAST with normal die surface. (**k**) Standard I_GSS_ I-V curve at V_g_ = 0 V to 6 V and V_d_ = 0 V before and after uHAST with abnormal die surface. (**l**) Standard I_DSS_ I-V curve at V_d_ = 0 V to 650 V and V_g_ = 0 V before and after uHAST with abnormal die surface.

In order to clarify the dielectric delamination and film cracking, an experimental design called the unbiased highly accelerated stress test (uHAST) is used to induce film delamination with high pressure and moisture, as shown in Figure 6c. For comparison purposes, we probe the fresh wafer with a standard I-V curve as shown in Figure 6a. The optical microscope check is performed after the fresh wafer characterization test to double confirm the wafer surface and document with images as shown in Figure 6b. The tested wafers are placed into a Teflon cassette and the uHAST test is run by following the JEDEC JESD22-A118 test condition A. The temperature is 130 °C, humidity is 85%, vapor pressure is 33.3 psi and test duration is 96 h, as shown in Figure 6c. Once the uHAST stress is completed, the wafers undergo a post-uHAST optical microscope check for surface abnormalities as shown in Figure 6e and run the post-uHAST I-V curve characterization as shown in Figure 6d. We compare the pre- and post-uHAST optical microscope check result and pre- and post-uHAST I-V curve characterization result as shown in Figure 6f to find delamination.

To illustrate the finding, we split the study into two subgroups by the die surface condition before and after uHAST. In Figure 6g, we look for dies that have a normal die surface before and after uHAST. Then, in Figure 6i, we compare the I-V curves before and after uHAST with a normal die surface. There is no difference for dies with a normal surface. In Figure 6j, we compare the I-V curves before and after uHAST with a normal die surface. The leakage increases by 10% after uHAST with a normal die surface. In Figure 6h, we look for dies that have an abnormal die surface before and after uHAST. Then, in Figure 6k, we compare the I-V curves before and after uHAST with abnormal die surfaces. There is no difference for dies with abnormal surfaces. In Figure 6l, we compare the I-V curves before and after uHAST with abnormal die surfaces. The leakage increases by 400% after uHAST with abnormal die surfaces. This finding is comparable with the die obtained from HTRB failure at 24 h.

**Figure 7:**
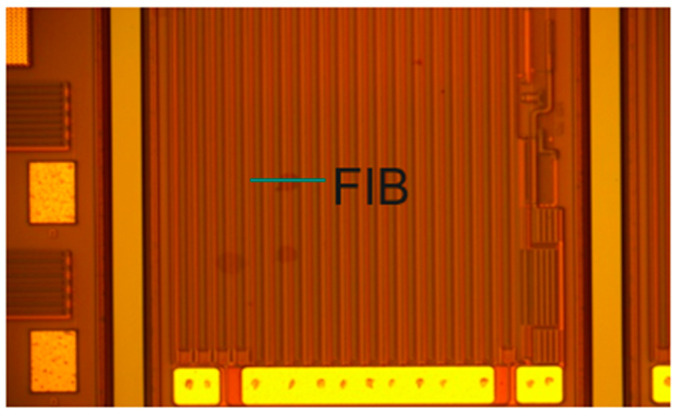
FIB cross-section check.

The finding from Figure 6l is consistent with what the HTRB fail part has shown after 24 h of stress. In Figure 7, it is a die with abnormal surface and a 400% increase in I_DSS_ leakage after uHAST. The FIB cross-section check is performed to identify the delamination.

**Figure 8:**
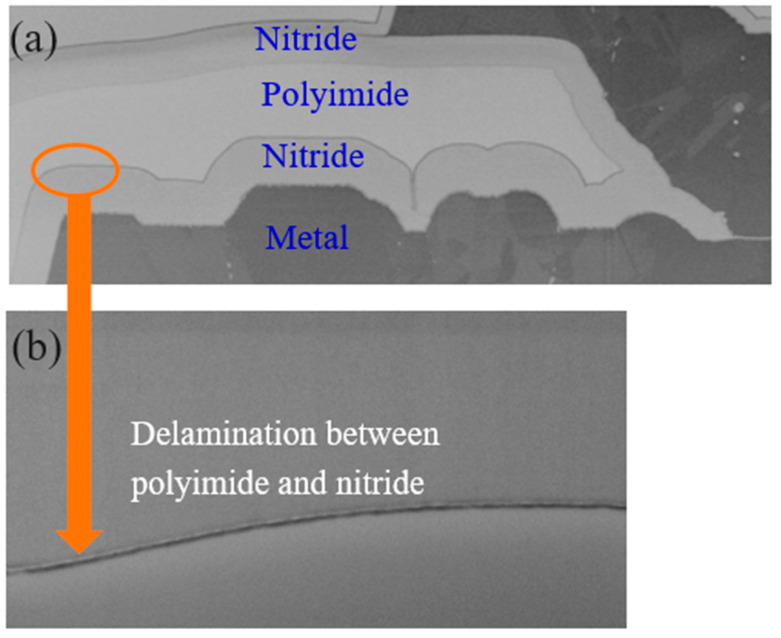
(**a**) Cross-section of the device with indicated delamination. (**b**) Enlargement of the delamination between polyimide and nitride.

The results from the cross-section checks on FIB are shown in Figure 8. In the default setting, the metal is bright and the dielectric is darker. It is difficult to find dielectric delamination. Therefore, reverse tone is used to make the metal dark and dielectric bright, as shown in Figure 8a. The delamination is found as the orange circle indicated and the same position with no reversing tone finds the delamination as shown in Figure 8b. We can clearly see the delamination between polyimide and nitride.

**Figure 9:**
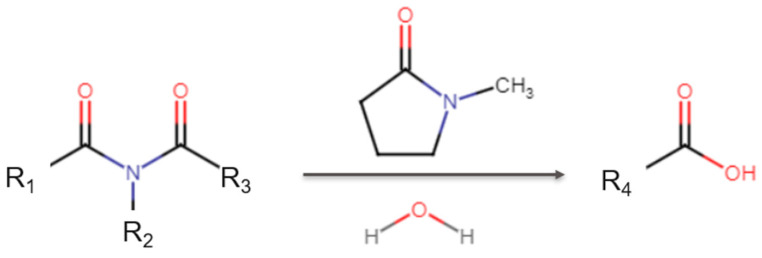
Chemical reaction of N-methyl-2-pyrrolidone (NMP) reacts with polyimide and water, forming carboxylic acid.

A detailed investigation into the processing steps and deposition recipes revealed the critical role of the N-methyl-2-pyrrolidone (NMP) cleaning procedure, which was applied both before nitride deposition and after polyimide deposition. This practice was found to be directly linked to the observed reliability failures. According to established chemical principles governing the reactivity of carboxylic acid derivatives, polyimides will react with NMP to form a corrosive carboxylic acid [9,10,11] (Figure 9). This reaction leaves a reactive and highly undesirable residue within the inter-dielectric layers, as illustrated in the cross-section shown in Figure 8b.

This residue fundamentally compromises the dielectric integrity, creating a latent and highly resistive leakage path. Under HTRB stress, this compromised region is subjected to both electrical and thermal loads, leading to accelerated heat accumulation and subsequent dielectric cracking and delamination, consistent with the findings shown in Figure 5a. This localized degradation path causes a significant increase in the off-state drain leakage current (I_DSS_), which initiates a positive feedback loop of thermal runaway, ultimately leading to catastrophic burnout at the drain terminal, as evidenced by the physical damage shown in Figure 5b.

Physical failure analysis of the failed die from the HTRB stress test further solidified this conclusion. Microscopic inspection revealed a distinct delamination between the polyimide and nitride layers, as depicted in Figure 5a, which precisely correlates with the delamination intentionally induced and observed in the separate uHAST experiments shown in Figure 8b. This strong correlation confirms that the chemical residue from the NMP cleaning process is the root cause of the observed delamination and subsequent catastrophic device failure under prolonged stress.

**Figure 10:**
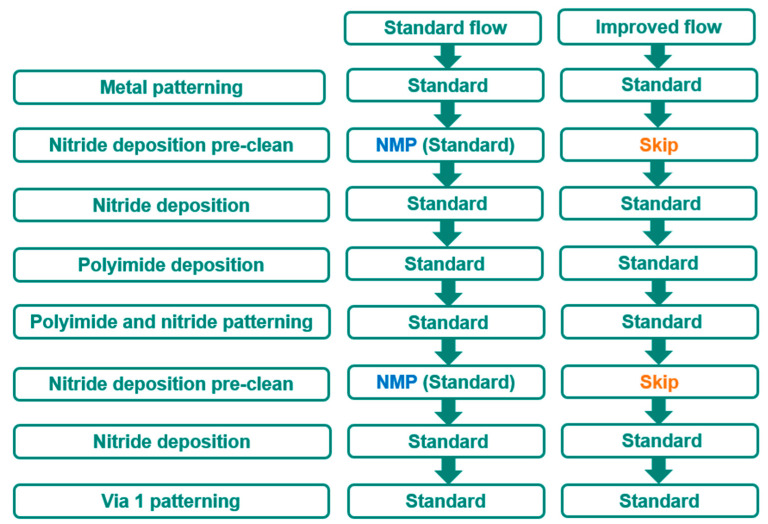
A brief process flow consisting of a standard practice causing a serious HTRB failure and an improved flow with skipping of two cleaning steps.

**Figure 11:**
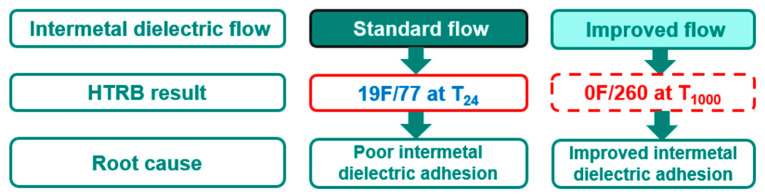
A summarized chart for the improvement of HTRB failures. The standard intermetal dielectric flow has 19 failures out of 77 parts due to intermetal dielectric film delamination. In the improved intermetal dielectric flow, there are no failures out of 260 parts after 1000 h of HTRB due to the improvement of intermetal dielectric adhesion.

After the removal of NMP cleaning from the standard process flow, as shown in Figure 10, three improved lots are evaluated. There is no failure out of 260 parts after 1000 h of HTRB. This result shows a significant improvement with no delamination and cracks after 1000 h of HTRB, as shown in Figure 11.

## 4. Conclusions

This study reveals a severe high-temperature reverse bias (HTRB) failure observed within 24 h of stress testing. Through a combined electrical and physical failure analysis, an underlying failure mechanism was identified where unbiased-highly accelerated stress testing (uHAST) was used to induce dielectric delamination. A strong correlation was established between delamination occurring at the nitride–polyimide interface and an increase in I_DSS_ leakage, exhibiting identical electrical performance. The subsequent removal of a suspected process step—specifically, the use of the chemical NMP before and after polyimide deposition—resulted in a significant improvement in failure rates. While initial HTRB failures occurred within 24 h, three lots totaling 260 parts sustained 1000 h of HTRB stress with zero failures. In summary, uHAST proved to be a valuable reliability testing tool for assessing package and film delamination stability in traditional reliability tests, but it can also be used as a troubleshooting methodology for other failures in reliability, such as HTRB. By leveraging high pressure and high moisture, this method effectively helps identify weaknesses in film adhesion and troubleshoot reliability-related failures.

## Data Availability

The datasets presented in this article are not readily available because [the data are part of an ongoing study or due to technical/time limitation]. Requests to access the datasets should be directed to Yu-Ting Chuang.
